# Method applied to the background analysis of energy data to be considered for the European Reference Life Cycle Database (ELCD)

**DOI:** 10.1186/s40064-015-0914-x

**Published:** 2015-03-28

**Authors:** Simone Fazio, Daniel Garraín, Fabrice Mathieux, Cristina De la Rúa, Marco Recchioni, Yolanda Lechón

**Affiliations:** European Commission, Joint Research Centre, Institute for Environment and Sustainability, Sustainability Assessment Unit, Via E. Fermi 2749 TP290, I-21027 Ispra, Italy; Spanish Ministry of Economy & Competitiveness CIEMAT (Research Centre on Energy, Environment & Tech) Energy Dpt. - Energy Systems Analysis Unit, Av. Complutense 40, E28040 Madrid, Spain

**Keywords:** LCI datasets, LCI database, Energy, Data quality, ELCD, PEF

## Abstract

Under the framework of the European Platform on Life Cycle Assessment, the European Reference Life-Cycle Database (ELCD - developed by the Joint Research Centre of the European Commission), provides core Life Cycle Inventory (LCI) data from front-running EU-level business associations and other sources. The ELCD contains energy-related data on power and fuels. This study describes the methods to be used for the quality analysis of energy data for European markets (available in third-party LC databases and from authoritative sources) that are, or could be, used in the context of the ELCD.

The methodology was developed and tested on the energy datasets most relevant for the EU context, derived from GaBi (the reference database used to derive datasets for the ELCD), Ecoinvent, E3 and Gemis. The criteria for the database selection were based on the availability of EU-related data, the inclusion of comprehensive datasets on energy products and services, and the general approval of the LCA community. The proposed approach was based on the quality indicators developed within the International Reference Life Cycle Data System (ILCD) Handbook, further refined to facilitate their use in the analysis of energy systems.

The overall Data Quality Rating (DQR) of the energy datasets can be calculated by summing up the quality rating (ranging from 1 to 5, where 1 represents very good, and 5 very poor quality) of each of the quality criteria indicators, divided by the total number of indicators considered. The quality of each dataset can be estimated for each indicator, and then compared with the different databases/sources. The results can be used to highlight the weaknesses of each dataset and can be used to guide further improvements to enhance the data quality with regard to the established criteria.

This paper describes the application of the methodology to two exemplary datasets, in order to show the potential of the methodological approach. The analysis helps LCA practitioners to evaluate the usefulness of the ELCD datasets for their purposes, and dataset developers and reviewers to derive information that will help improve the overall DQR of databases.

## Introduction

After its debut in the European Commission’s Integrated Product Policy (European Commission 2003) as the “best framework for assessing the potential environmental impacts of products”, life cycle assessment (LCA) has been increasingly used to support EU policies. Since then, the use of LCA and life cycle approaches has been progressively advocated and/or adopted in a wide range of European Commission policies.

In the context of Europe’s 2020 Flagship Initiative “A Resource Efficient Europe” (European Commission 2011a), the European Commission issued a Communication on Building the Single Market for Green Products and a Recommendation on the use of the methods (European Commission 2013a), in which it developed its recommendations for the Product Environmental Footprint (PEF) and the Organisation Environmental Footprint (OEF) methodologies (European Commission 2013b). These methodologies represent a vital milestone towards increasing the coherence and quality of assessments of the environmental performance of products and organisations by governments and business stakeholders. Life Cycle Thinking is essential in modern decision making for business and policy. Commonly implemented through LCA, it is increasingly necessary to help quantify the benefits and burdens that occur in the supply chains, use, and end-of-life of products (both goods and services).

Within this framework, the European Platform on Life Cycle Assessment (EPLCA 2014), developed by the Joint Research Centre (JRC) of the European Commission and DG Environment, represents the reference point for data and methods that are essential to implementing life-cycle-based approaches. While the information contained within the EPLCA relates mainly to the EU, it is extending its reach to the global level (several international contributors have already joined, and others are about to join). The Platform promotes the availability of data and information, with a focus on coherence and quality assurance. Although the methodology is developing at a fast pace, particularly with regard to the requirements of the European Commission, the lack of coherent, quality-assured life cycle data and studies still represents a major challenge to the mainstream use of LCA and associated environmental footprint methods in the business and policy sectors. Since its first release in 2006, the European Life Cycle Database (ELCD, developed under the EPLCA framework and managed by the JRC) gathers life cycle inventory (LCI) data from EU business associations (members of the advisory group of the EPLCA) and other sources for information on key materials, energy carriers, transport, and waste management. Most of the respective datasets are officially provided and approved by the industry associations involved. Many datasets have been revised and reviewed against the ILCD’s Entry-Level Requirements (European Commission JRC, Institute for Environment and Sustainability (2010a)), and others are currently under review. This enabled the ELCD 3.0 (ELCD 2014) database (launched in 2013) to become one of the first nodes of the Life Cycle Data Network (LCDN 2014; Recchioni et al. 2014), which was officially launched in February 2014. The datasets were reviewed against the ILCD Entry-Level Requirements in order to provide users with useful information on data quality, including minimum documentation and methodological consistency across datasets, and to ensure the availability of reliable data to be used in LCA studies. In a spirit of continuous improvement, the JRC will carry out a background analysis of key (energy) datasets of the ELCD in order to identify opportunities for improving the quality of the data. This paper describes the methodological approach that was used to carry out the analysis of the data quality of the energy datasets included in the current version of the ELCD. This method does not aim to compare the overall quality of existing databases, but to point out the possible strengths of third-party databases with regard to data quality ratings (DQRs), in order to improve the quality of datasets included in the ELCD. Although the DQR evaluation, which was carried out against the ILCD Handbook criteria, can lead to lower average DQR scores for third-party databases that are compiled using different approaches, particular strong points in terms of data quality can be highlighted and used as an example for the improvement of the ELCD.

The paper is structured as follows: Section State-of-the-art Data Quality of LCI Datasets and Energy Datasets summarises the literature on energy datasets and data quality; Section Proposed method introduces the main aspects of the developed method; Section Application of the method to exemplary datasets illustrates the application of the method to two exemplary datasets; Section Discussion and Conclusions discusses the validity of the method based on the preliminary results, and draws conclusions.

## State-of-the-art data quality of LCI datasets and energy datasets

### Importance of quality-assured LCI energy datasets

Although LCA-based methodologies and tools seem to develop quickly, the availability of quality-assured LCA data still represents a major bottleneck to the broader use of LCA and environmental footprint methods in the business and policy sectors.

In LCT and LCA approaches, the quality of the so-called secondary data, and particularly those related to the energy sectors (power and fuels), are deemed to be highly relevant in order to obtain consistent results. Lack of adequate quality data can, in fact, adversely affect the repeatability, reliability and comparability of LCA (Bjorklund 2002).

Electricity and fuels are commonly used in the production, transport and use phase of products and services. Also the existing international standards requiring complete and representative LCI dataas regards the energy sector. For instance, ISO 14040–44 (ISO 2006a, b) states that “*When determining the elementary flows associated with production, the actual production mix should be used whenever possible, in order to reflect the various types of resources that are consumed… …for the production and delivery of electricity, account shall be taken of the electricity mix, the efficiencies of fuel combustion, conversion, transmission and distribution losses*”. LCI data related to energy sectors (i.e. power and fuels), as well as some secondary data (e.g. raw material commodities, end-of-life scenarios) are considered to be highly relevant to LCA results, in terms of environmental impacts and resource consumption (De Smet and Stalmans 1996; Treyer and Bauer 2013). The quality, consistency and representativeness of these data are therefore crucial (Treyer and Bauer 2013).

In its “Specification for the assessment of the life cycle of Greenhouse Gas Emissions of goods and services” (BSI 2011), the British Standards Institution includes the following guideline: “*for electricity and heat delivered via a larger energy transmission system, secondary data that is as specific to the product system as possible (e.g. average electricity supply emission factor for the country in which the electricity is used) should be used*”. This highlights the need for transparent and high quality energy datasets in LCA or related methods.

### Methods for assessing the data quality of LCI datasets

In order to be compliant with ISO 14044, section 4.2.3.6 (ISO 2006a, b), LCI datasets must include a data quality description of its time-related, geographic and technological representativeness as well as of the precision, completeness, consistency and uncertainty of the information. Under this framework, some guidelines have been developed to address the DQRs. For instance, in the ILCD Handbook (European Commission JRC and Sustainability 2010c), data quality scores rank the datasets based on six indicators (technological representativeness, geographical representativeness, time-related representativeness, completeness, precision/uncertainty, and methodological appropriateness and consistency) with data quality scores of 1 to 5 (high to low) assigned to each.

Similarly, in the framework of the UNEP/SETAC Life Cycle Initiative (UNEP - SETAC Life Cycle Initiative 2011), the Global Guidance Principles for LCA databases refers to the Data Quality Indicators of ISO 14040–44, including those scored using the ILCD method, as well as reproducibility, representativeness, and information on data sources (the latter are not included in the ISO guidelines).

The U.S. LCI Database Project Development Guidelines (ATHENA 2004) describes data quality based on the age, source and collection method of the data; data representativeness (e.g. the percentage of total production represented by a sample); averaging methods; methods used to estimate or justify data gaps; and information about key assumptions or methodological choices. Information on uncertainty is not required, so, for example, data derived from statistics provided by authoritative sources are considered to be at the same level as a single data point from a personal communication.

The “pedigree matrix” is a specific DQR proposed in the database Ecoinvent (Weidema et al. 2011), where data quality scores are developed using a five-point scale, similar (in terms of aspects considered) to the ILCD method, but at the emission level of the data. The data scores take into account aspects such as geographical, technological and temporal validity, the origin, representativeness and validation of the data, and administrative information. Ecoinvent was one of the first data developers dealing with the DQ issues, the process started more than 10 years ago (Frischneckt et al. 2005).

### Towards a more sectorial definition of data quality

It is quite difficult to differentiate between ‘good’ and ‘poor’ quality of LCI datasets, since several data quality requirements can be defined only in relation to the specific study or context in which they are used. Furthermore, data quality is often related to the analyst’s degree of knowledge of the product or process being analysed. The review of the dataset and the quality assurance of the data should therefore be sector-specific, and the data quality indicators should consequently be adapted/refined at the sector level (Bellon-Maurel et al. 2014; De Smet and Stalmans 1996; Lasvaux et al. 2014; Maurice et al. 2000). May and Brennan (2003a, b) applied six different data quality assessment methods to a case study of LCA for electricity generation and showed that the qualitative evaluation methods presented significant data quality information, allowing for the identification of the source and type of deficiencies in data quality. Their papers also underlined the need for further sector-specific improvements in qualitative assessment methods.

Various authors have carried out cross comparisons of LCI databases; for example, Lesage and Samson (2013) show how several ‘foreign LCI databases’ were analysed and cross-compared using various criteria in order to identify the most appropriate database to use as a basis for the Quebec LCI database.

Within this framework, the sector-specific adaptation of the data quality assessment method proposed in the ILCD Handbook for the energy sector, and the cross comparison of databases were deemed to be the best choices for the targeted improvement of the ELCD.

## Proposed method

### Context and overview of the method

**Figure 1:**
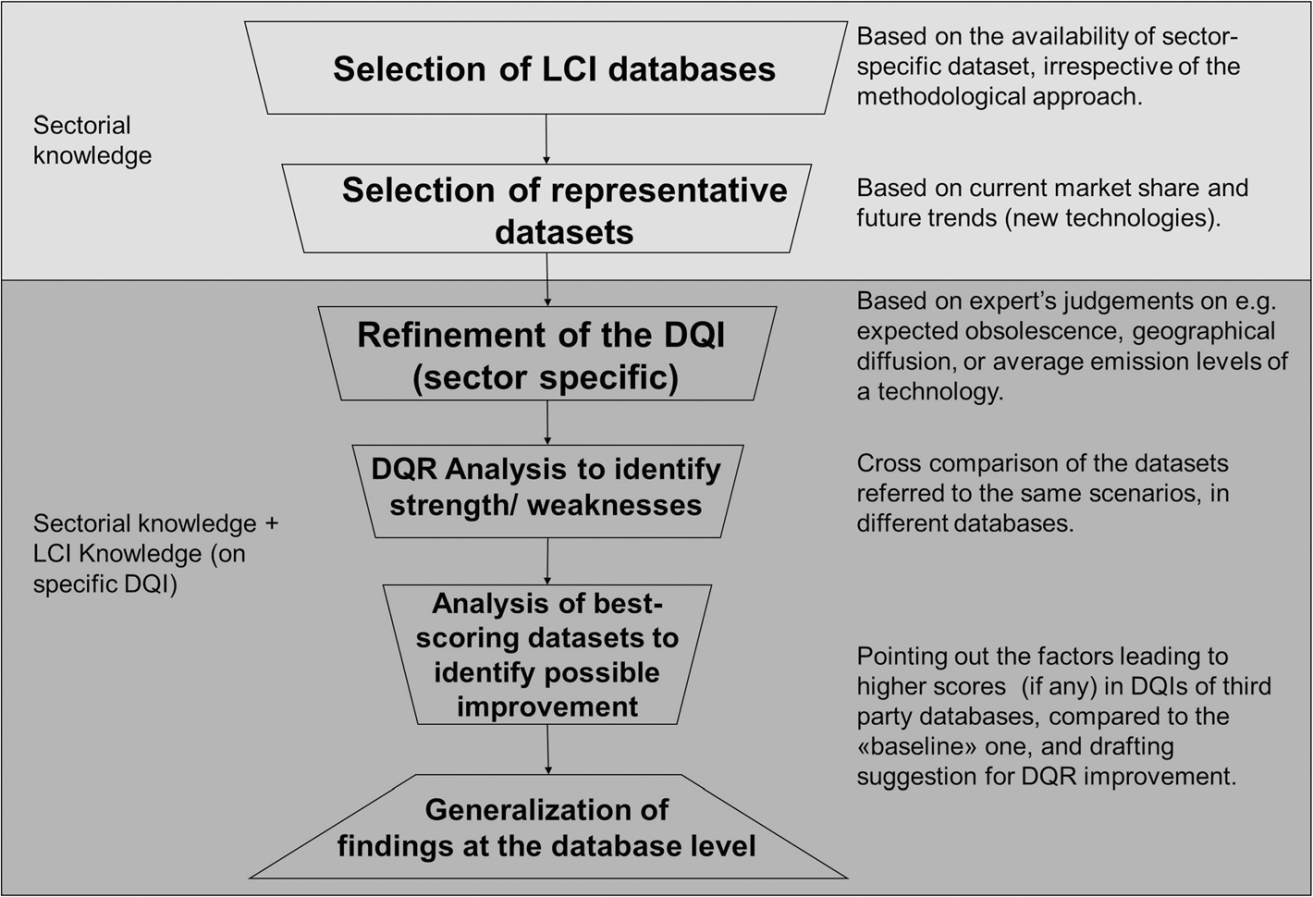
**Methodological steps for the background analysis of energy datasets.**

As highlighted in the introduction and in the literature review, current LCA applications require the data quality of LCI datasets to be transparently documented and to fulfil specific requirements. Since data quality issues in LCI databases are still debated and being continuously improved, a regular review of datasets should also be carried out in order to improve the overall DQR. Therefore, the JRC decided to lead a background analysis of the energy datasets included in the ELCD, in order to identify weaknesses and opportunities for improvement of data quality. The evaluation was carried out by the JRC’s Institute for Environment and Sustainability together with the Energy Systems Analysis Unit of the Spanish Research Centre for Energy, Environment & Technology (CIEMAT). Since no workable method for carrying out this background analysis was identified in the literature, a method was developed. This new method, which is presented in this section, is composed of six steps that are summarised in Figure 1. Steps 1 to 3 are presented in detail in the following sections, while steps 4–6 are described in the following chapter.

### Analysis and selection of LCI databases

The aim of the proposed method is to benchmark the data quality of the dataset or group of datasets being studied against similar datasets in other LCI databases. To do so, databases that are relevant to the specific sector must be identified in advance.

The databases to be analysed together with the ELCD have been chosen based on their of energy datasets. The Ecoinvent database (Ecoinvent 2013) was chosen as it is considered to be one of the most widely used LCI databases with a general background, and includes a broad range of energy and fuels datasets. GaBi (PE 2013), another major LCI database, was not selected because ELCD energy datasets are actually based on GaBi datasets and hence a cross analysis would not bring any benefits. Two other energy-oriented databases (GEMIS (IINAS 2013) and E3 (LBST 2013)) were chosen to complement the selection. The selection of databases was made irrespective of their methodological compliance with the ILCD quality criteria: it was assumed that although other databases might have lower DQR scores based on ILCD rules (because they were not specifically developed using these rules), they could represent interesting benchmarks from which some improvement to data quality could be derived.

### Analysis and selection of energy datasets

Several authors have highlighted the importance of performing a pre-analysis in order to evaluate the representativeness, in terms of geographical origin and market share (current or future), of the energy datasets on which the background analysis is being carried out, both for fuels and electricity. The use of authoritative sources for this pre-analysis has been widely recommended (Di et al. 2007; Itten et al. 2014; Treyer and Bauer 2013, 2014).

The evaluation of data quality against the ILCD requirements (both for the entry level and full compliance) cannot be automated as it requires expert judgements and critical evaluation of the information provided. Therefore, and in order to keep the method workable with limited resources, the selection of a representative (i.e. in terms of market share, not simply statistically) sample of datasets to be analysed is a pre-requisite for a background analysis of entire databases (those used for the validation of the method are listed in Table 1). As energy datasets are often used as a secondary data source, the selection was made taking into account the current market share of electricity and fuels in the EU, as well as future projections for renewable energy solutions that are expected to become relevant in the near future. The most representative (currently and for the future) energy datasets were chosen from the selection based on the following (derived from authoritative sources):

- Related to electricity: datasets representing a significant share (i.e. based on expert judgement) of the EU electricity market, considering also associated technology mixes and geographic origins; for the analysis it was decided, based on expert judgement, that the sum of studied technology should represent 40 to 60% of the EU market;
- Related to fuels: representative datasets of crude oil and natural gas that are relevant to the EU market, based on expert judgement, with different percentages depending on the fuel/technology considered;
- Other considerations support the inclusion of some minority energy sources such as some renewable sources whose contribution to the European energy mix is likely to become more important in the future, based on forecasting models.

**Table 1:** **List of the selected energy datasets used in the analysis of ELCD datasets when benchmarking them with datasets from three other databases**

**Category**		**Location**	**Name of LCI process**
Electricity	Mix	EU-27	Electricity grid mix (1 kV - 60 kV)
	Coal	DE	DE: Electricity from hard coal (1 kV - 60 kV)
		GB	GB: Electricity from hard coal (1 kV - 60 kV)
		PL	PL: Electricity from hard coal (1 kV - 60 kV)
	Lignite	DE	DE: Electricity from lignite (1 kV - 60 kV)
		GR	GR: Electricity from lignite (1 kV - 60 kV)
		PL	PL: Electricity from lignite (1 kV - 60 kV)
		CZ	CZ: Electricity from lignite (1 kV - 60 kV)
	Natural gas	GB	GB: Electricity from natural gas (1 kV - 60 kV)
		IT	IT: Electricity from natural gas (1 kV - 60 kV)
		DE	DE: Electricity from natural gas (1 kV - 60 kV)
		ES	ES: Electricity from natural gas (1 kV - 60 kV)
	Nuclear	FR	FR: Electricity from nuclear (1 kV - 60 kV)
		DE	DE: Electricity from nuclear (1 kV - 60 kV)
	Hydro	EU-27	Electricity from hydro power (1 kV - 60 kV)
	Wind	RER	Electricity from wind power (1 kV - 60 kV)
	Biomass	DE	DE: Electricity from biomass (solid) (1 kV - 60 kV)
	Solar	DE	DE: Electricity from photovoltaic (1 kV - 60 kV)
Crude oil and natural gas based fuels		EU-27	Diesel mix at refinery
		EU-27	Gasoline mix (regular) at refinery
		EU-27	Heavy fuel oil at refinery (1.0wt.% S)
		EU-27	Kerosene/Jet A1 at refinery
		EU-27	Natural gas mix
Biofuels		DE	DE: Rapeseed Methyl Ester (RME)

According to European statistics (Eurostat 2013; MOE 2011), the most representative power generation technologies in 2011 for the EU were the following: Nuclear (27%), Coal (26%), Gas (23%), Hydro (13%) and Wind (4%); all of these were selected for the analysis. Other renewable energy sources make a lower contribution to electricity generation in the EU, such as biomass and waste, and solar energy (3% and 0.68%, respectively). However, due to their foreseen potential, their contribution is expected to increase in the future (Arvizu 2010; European Commission 2011b), in order to meet the future energy demands without increasing greenhouse gas (GHG) emissions (Luque 2008).

The electricity generated by the aforementioned sources was considered for the analysis. The EU’s electricity mix was also taken into account, even though the ELCD does not allow the unit processes used to build the datasets to be broken down into technologies, which limits the analysis of data quality. However, given that the ELCD energy datasets originated from the GaBi Database (PE 2013), which allows for the analysis of energy mixes by technology, specific GaBi datasets were analysed whenever they were not available in the ELCD. A drawback of the GaBi Database for this analysis is that it does not include datasets for each technology specific to the European context, i.e. electricity production from hard coal and the European electricity mix. Therefore, in order to take into account the European energy market, datasets were chosen from the GaBi Database only for those countries that account for 60% of the electricity produced in Europe for each technology (derived from Eurostat (2013), based on data from 2010).

European statistics on fuel production (MOE 2011) show that the most representative oil derivatives produced in Europe were the following: Diesel (around 37% of refinery output), Gasoline (20%), Residual fuel oil (15%) and Kerosene (6%). Due to their relevance in the share of fuel production, these products were considered for the analysis.

Additionally, an analysis of the gross amount of heat generation in the EU (Eurostat 2013) found that natural gas is the largest source of fuel, with a contribution to heat generation of around 44%; natural gas datasets were therefore also taken into account in the current analysis. Although the market share of biofuels is still barely significant with respect to total fuel consumption, biofuel production has significantly increased during the past decade due to a favourable framework and the support of several policies. Europe’s contribution to biofuel production is foreseen to increase in the coming decades. As rapeseed oil seems to be one of the raw materials expected to significantly contribute to the production of biodiesel, Rapeseed Methyl Esther (RME) was also included in the analysis (MOE 2011).

### Refinement of Data Quality Indicators (DQIs)

The proposed evaluation was based on the quality indicators developed for and included in the ILCD Handbook (European Commission JRC, Institute for Environment and Sustainability 2010a, b, c). Considering the purpose of the study, the following criteria were considered and refined when necessary (the scores are summarised in Table 2):

- Technological (TeR), Geographical (GR) and Time-related (TiR) representativeness: datasets related to the most representative energy technologies in each area, within the European market context, based on the abovementioned criteria derived from authoritative sources. The origin of any raw fuels imported for power and fuel production were listed for each chosen country. TiR related to the expected obsolescence of the technology applied (based on existing data) defined as the year(s) in which the data were collected, with a deviation of ±5 years. The framework is the same as that proposed by the ILCD Handbook, and sector-specific expert judgement was used to define the criteria (e.g. the adjustment on elementary flows coverage, quality of references, etc.).
- Completeness (C): defined in ISO and ILCD as the share of elementary flows (i.e. the percentage of relevant flows in terms of relevance in the impact assessment), weighted against the percentage of environmental impact categories that are quantitatively included in the inventory. A pre-analysis based on sectorial experience was carried out to identify the elementary flows (including a list of those most relevant based on mass and/or impact) that allow for the estimation of the 16 environmental impact categories at the mid-point level in the method recommended by the ILCD (see European Commission 2011a, b, for an extended review). In this study, the preliminary analysis of completeness led to scores based on the number of impact categories covered, which were then adjusted in relation to the coverage of relevant elementary flows included in the dataset (i.e. no changes if the flow list included more than 75% of the flows, one level lower if the flow list only covered 50 to 75%, and two levels lower if the flow coverage was less than 50%).
- Precision/uncertainty (P): both the reliability of the data and degree of uncertainty of the information (data, models and assumptions) were accounted for. An expert judgement was made based on the quality of the references and their sources, whether measured, calculated or estimated from the literature.
- Methodological appropriateness and consistency (M): To evaluate this criterion, the LCI modelling framework and LCI method approaches recommended by the ILCD Handbook were correctly and consistently applied for each situation, focusing on three issues: i) System boundaries; ii) End-of-Life (EoL) modelling; and iii) Multifunctionality (in line with the different contexts defined in the ILCD Handbook).

**Table 2:** **Matrix for assessing data quality of datasets as proposed in the ILCD Handbook in italic the sector-specific refinements/judgements, leading to the definition of DQI ranges**

**Indi-cator**	**Subquality parameters**	**Rating**				
		**1 (Very good)**	**2 (Good)**	**3 (Fair)**	**4 (Poor)**	**5 (Very poor)**
**TeR**	Expert judgement (*technology mix* (t.mix))	modelled as the t.mix	very similar to the t.mix	similar to the t.mix	different to the t.mix	totally different to the t.mix or not assessed
**GR**	Expert judgement (*share of referenced countries* (r.c.))	Fulfil totally the share of r.c.	Fulfil very similarly the share of r.c.	fulfil similarly the share of r.c.	Fulfil differ-rently the share of r.c	Fulfil completely different the share r.c.
**TiR**	Expert judgement (*defined time* (d.t.) on data inventory)	All the data sources refer to d.t.	The majority of the data sources refer to the d.t.	At least half of the data sources refer to the d.t.	Less than half of data sources refer to the d.t.	None the data sources refer to the d.t.
**C**	No. of impact categories *weighted on% of elementary flows*	15-16 impact categories	12-14 impact categories	8-11 impact categories	5-7 impact categories	≤5 impact categories
**P**	Expert judgement *precision/uncertainty (p/u) of data sources*	Very low u. and/or very high p.	Low u. and/or high p.	Fair u. and/or fair p.	High u. and/or low p.	Very high u. and/or very low p.
**M**	expert judgement (*system boundaries, multifunctionality and EoL*) based on the situation (European Commission JRC, Institute for Environment and Sustainability 2010a), completion (C.) degree	all LCA stag-es, allocation procedures. C. in a very high degree	Most relevant LCA stages. allocation procedures. C. in a high degree	Sufficient LCA stages. allocation procedures. C. in a suffi-cient degree	Sufficient LCA stages. allocation procedures. C. in a low degree	Not sufficient LCA stages. No allocation procedures (multi-functionality not solved according to the situation) C. in a low degree

All the information related to the datasets was derived from the documentation attached to each dataset and integrated, where needed, with extra information provided through confidential reports, by the database developers.

## Application of the method to exemplary datasets

In order to illustrate the method and possible results, this section reports its application to two exemplary datasets of the ELCD - nuclear power generation for France, and the diesel mix at the EU level. Of course, opportunities for improvement cannot be prioritised nor can conclusions be drawn at the database level based on the analysis of the two datasets, as proposed in Figure 1. However, more exhaustive results and a discussion at the database level are in press (Garraín D. et al. Background qualitative analysis of the European Reference Life Cycle Database (ELCD energy datasets – Part I: Fuel datasets, *Springer Plus* (in press) and Garraín D. et al. Background qualitative analysis of the European Reference Life Cycle Database (ELCD) energy datasets – Part II: Electrocity datasets, *Springer Plus* (in press)).

The analysis is based on a benchmarking of the ELCD datasets against similar datasets extracted from other third-party databases such as Ecoinvent (Ecoinvent 2013) Gemis (IINAS) and E3 (LBST). The specific datasets were chosen as they are the most representative within their respective technologies. In the case of diesel mix, the ELCD achieved the best score in all the DQIs, while other third-party databases scored better than the ELCD in two DQIs of the nuclear power scenario. The different ranking can better explain the potential benefits that can be derived from the background analysis, taking into account the improvements that led to a better score in other databases.

### Application to a nuclear electricity dataset

In general, nuclear electricity datasets in the ELCD have a lower DQR score (i.e. higher DQR = lower score) than fossil-fuel-generated electricity datasets (for which the ELCD datasets generally achieved the highest DQRs), and other analysed databases perform better on other criteria (see Table 3 for a complete list of the scores of the analysed datasets, and a short explanation of the judgements on which they were based).

**Table 3:** **DQRs of the exemplary dataset, under the different databases**

**Dataset**	**Database**	**DQI**	**Score**	**Short justification of DQI**	**DQR**
**Electricity From nuclear power (FR)**	ELCD	TeR	1	Modelled as the French technology mix	1.83
		GR	2	Some activities of milling and reprocessing refers to US data	
		TiR	3	Some references are 20 years older than the ref. year (2009)	
		C	1	100% of impact categories and 100% of reference flows covered	
		P	2	Relevant flows measured, other flows taken from literature	
		M	2	EoL of intermediate activities is missing	
	Ecoinvent	TeR	2	Some data extrapolated from Swiss power plants	1.67
		GR	2	Infrastructure data from Swiss plants, only 1 uranium supplier	
		TiR	2	Ref. year 2002, relevant data are more updated than ELCD	
		C	1	100% of impact categories and 100% of reference flows covered	
		P	2	Relevant flows measured, other flows taken from literature	
		M	1	EoL and allocation also for sub-processes	
	GEMIS	TeR	2	Referred to French representative plants but not as a mix	3.08
		GR	4	Only the modeling of enrichment is correct	
		TiR	2-3	(depending on plant) literature comes from 5–15 years before	
		C	2	75% of impact categories, 90% of flows covered	
		P	4	Literature data and auto-estimated data	
		M	4	EoL not modeled, not including infrastructures.	
	E3	TeR	4	Considering a process scale instead of real plant	4.00
		GR	4	Only the modeling of enrichment is correct	
		TiR	3	Reference year 2000, data from 1994-99	
		C	4	Less than 50% impact categories, 90% flows covered	
		P	4	Literature data and auto-estimated data	
		M	5	Cradle to gate system, EoL and infrastructure lacking	
**Diesel mix (EU27)**	ELCD	TeR	1	Relevant primary and secondary data referred to EU27	1.08
		GR	1	Very good modeling of EU27 share and market relevance	
		TiR	1	Ref year 2009, data from 2007 to 2009	
		C	1	100% of impact categories, 96% of flows covered	
		P	1-2	Some data are calculated basing on technical descriptions	
		M	1	Cradle to grave process, EoL and infrastructure included	
	Ecoinvent	TeR	2	Some transport distances refers to Swiss refineries	1.75
		GR	2	Few countries not included	
		TiR	1-2	Ref year 2000, some data from ‘80s	
		C	1	100% of impact categories and 100% of reference flows covered	
		P	2	Some oil extraction data from Africa are roughly estimated	
		M	2	EoL not modelled, infrastructure included	
	GEMIS	TeR	3	Modelled by a generic plant, default distance values	3.50
		GR	5	Not referred to any specific country	
		TiR	4	Ref year 2000, data from 1985-95	
		C	2	75% of impact categories, 90% of flows covered	
		P	4	Estimated data from literature, assumptions not disclosed	
		M	3	EoL not comprised, Allocation not specified	
	E3	TeR	2	Modelled from CONCAWE report assuming oil from middle east	2.67
		GR	3	Extraction only from mid. east, representativeness of EU refinery system is not explained	
		TiR	2	Ref. year 2010, data coming from CONCAWE (1996–2007)	
		C	4	Less than 50% of impact categories, 90% of flows covered	
		P	2	No info about emission factors	
		M	3	Cradle to gate system, EoL not included.	

Table 1 lists the datasets that were chosen as the basis for the comparison of databases and with other potential sources, in order to improve the ELCD’s overall DQR.

It is important to highlight that the DQRs presented in this Section (in Table 3) were calculated using a slightly adapted ILCD method. As shown in Section State-of-the-art Data Quality of LCI Datasets and Energy Datasets, several DQR systems exist, and all of the third-party databases analysed use their own system, not that of the ILCD (used for ELCD). It is therefore no surprise if ELCD datasets behave well within such a system, while others do not. Recalling the context of the analysis and the objectives of the method presented in Section Context and overview of the method, the results presented here do not represent a suggestion for the use of a specific database, but they are only useful to identify relevant improvement opportunities for the DQIs (and hence the DQRs) of ELCD datasets, and ultimately to improve the quality of the ELCD.

In the chosen datasets on the electricity from nuclear power in France, Ecoinvent performs better than the ELCD in the TiR category since the validity period of the dataset is closer to the oldest references, and in the M criterion since it considers a final repository of spent fuel and high-activity waste that is not included in the ELCD.

As shown in Table 3, the ELCD scores worst on the TiR category, specifically for the chosen dataset. The reason lies in the use of several old references. However, no better references could be found in the other databases analysed. Those available in Ecoinvent refer to the same timescale, but the declared year of reference (i.e. the time slot referred to) of the dataset is closer to the reference years considered (i.e. the time reference of the data collection), which makes the TiR more consistent even if slightly outdated. As regards the M criterion, the better score of Ecoinvent also highlights the need to improve the end-of-life analysis for background processes and sub processes.

The ELCD uses the work of Dones and Zollinger (1996) as an important reference. This work was significantly updated in Dones et al. (2007) with an improvement identified in the Ecoinvent database. Some data for the enrichment state derived from the earlier report can be updated with data from the 2007 one.

The geographical representativeness of the ELCD dataset could be improved upon using data from Canadian mines and mills (which are closer to the real French scenario, rather than the currently used US data which are primarily responsible for the underscoring from 1 to 2) that can be obtained for example from CERI (2008) or UNSCEAR (2000).

The methodology score can be improved by including a final repository (i.e. the end-of life (EoL) criterion mentioned in Table 3) for spent fuel and nuclear waste using data from NAGRA (Nagra 2002a, b).

### Application to a diesel mix scenario dataset

An analysis of the DQR of the diesel mix scenario showed that, even though the ELCD’s overall score for all criteria considered is higher and the completeness scores of both datasets are the same, the completeness of flows covered is actually higher in the Ecoinvent dataset. It emerged that some flows are missing from the ELCD datasets, such as CFC-11 and CFC-12 for the Ozone Depletion category, and the Decane emission for the freshwater ecotoxicity category, but are included in Ecoinvent. This is a clear demonstration of the fact that human interpretation of the analysis is needed to identify possibilities for improvement of the ELCD, even where the DQI rankings are similar.

## Discussion and conclusions

The method developed could be very useful for helping to improve datasets through cross-analysis and comparison of single DQIs and overall DQRs. The approach could also be very useful both for evaluating the quality of a single database, and for assessing the overall quality of entire or partial databases.

The results can be analysed both horizontally (i.e. taking into account the same DQI, related to the same dataset from different databases, pointing out the possibility for improvement), or vertically (i.e. analysing the overall DQR of a dataset within a single database, highlighting the strong and/or weak points in terms of scores within a single dataset, by group of technologies, sectors or databases). In both cases the DQI and DQR analysis only represents a starting point for the improvement of data quality; the key steps for identifying possible enhancements can only be made using expert judgement. In LCA, the quality of a dataset or database should be evaluated in such a way that the final conclusions derived from the use of the dataset are sufficiently robust and are in line with the goal and scope described in the metadata. The robustness should be ensured by the use of datasets, in which the technology, the time horizon defined and the geographical area considered are appropriate to model the system according to the goal and scope of the study. Furthermore, it should be ensured that the data used to build the dataset properly describe the relevant inputs and outputs (considering uncertainties due to measurements, process-specific variations, temporal variations), that the elementary flows included cover the most relevant impacts, and that the methodology used to build the dataset is appropriate to model the analysed system.

This paper presents an original and workable (i.e. assuming a fixed amount of resources and time) methodological approach that analyses the data quality of a group of datasets of a given database, by cross-comparing them with datasets from other databases, and identifies opportunities for improvement of the datasets/database under scrutiny. The six quality criteria indicators defined by the ILCD Handbook have been included in the method. These indicators have, however, been redefined in order to facilitate their implementation, and to ensure the quality of the assessment whenever expert judgement was required. The quality criteria indicators can be applied to any type of LCA dataset. However, in order to ensure the appropriateness and robustness of the methodology applied, in-depth knowledge of the analysed topic is required, since expert judgement values have been applied in many cases.

The methodology used for the background analysis of energy data to be considered for the ELCD is an example of the sectorial application of the general ILCD guidelines related to data quality assessment. It can be concluded that the general framework outlined within the ILCD Handbook needs to be adapted on a sectorial basis, in order to ensure the appropriateness of the DQR. This is in line with existing literature. Expert judgements are therefore required not only in the evaluation phase but also in the definition of the data quality evaluation methods and criteria. Taking these considerations into account, data quality assessments conducted using the proposed method should not be extrapolated to datasets under different contexts.

The cross comparison of datasets from different databases that refer to the same technology can lead to the identification of key aspects to be improved in order to enhance the overall quality of data. As shown in the examples, this is possible even if different datasets achieve the same ranking in a specific DQI. Furthermore, the analysis of a single dataset, or homogeneous groups of datasets in the same database, can highlight areas to be improved, focusing on specific weak points at dataset or database level (if there are common DQIs to be improved across different datasets). The approach adopted in the development of the proposed method (i.e. the state-of-the-art analysis, followed by the choice of representative datasets and databases, and the selection and adjustment of the criteria for the DQR assessment) can be applied to different sectors and product categories, with the advice of sector-specific experts. The time span for the future revisions can also be decided during the state-of-the-art analysis, depending on, for example, the expected obsolescence of technologies, or the forecasts of changes in market share. The accurate selection of the dataset is also deemed to be extremely important, as it is key to the representativeness of the results of the analysis.

The method can be also implemented for the partial analysis of datasets from other sectors that are strongly affected by the consumption of power and fuels, such as transport and some industrial sectors.

Generally speaking, a sector-specific method for the background analysis of data can be useful both for database developers and for reviewers. The former will benefit from such analyses in order to improve their datasets and to focus their efforts on the improvement of specific DQIs, and the latter could benefit from a more detailed evaluation framework that highlights the hotspots that can significantly affect the overall DQR in a specific sector. For LCA practitioners the benefits to be derived from such an approach include the overall improvement of data quality, consistency, and reliability.
